# Toxic metals impact gut microbiota and metabolic risk in five African-origin populations

**DOI:** 10.1080/29933935.2025.2481442

**Published:** 2025-04-09

**Authors:** Julianne A. Jorgensen, Candice Choo-Kang, Luyu Wang, Lina Issa, Jack A. Gilbert, Gertrude Ecklu-Mensah, Amy Luke, Kweku Bedu-Addo, Terrence Forrester, Pascal Bovet, Estelle V. Lambert, Dale Rae, Maria Argos, Tanika N. Kelly, Robert M. Sargis, Lara R. Dugas, Yang Dai, Brian T. Layden

**Affiliations:** a Division of Endocrinology, Department of Medicine, University of Illinois Chicago, Chicago, IL, USA; b Department of Bioengineering, College of Engineering, University of Illinois Chicago, Chicago, IL, USA; c Public Health Sciences, Parkinson School of Health Sciences and Public Health, Loyola University Chicago, Maywood, IL, USA; d Department of Pediatrics, Center for Microbiome Innovation, University of California San Diego, La Jolla, CA, USA; e Department of Physiology, School of Medicine and Dentistry, College of Health Sciences, Kwame Nkrumah University of Science and Technology, Kumasi, Ghana; f Solutions for Developing Countries, University of the West Indies, Mona, Kingston, Jamaica; g Department of Epidemiology and Health Services, University Center for General Medicine and Public Health (Unisanté), Lausanne, Switzerland; h Ministry of Health, Mahé, Victoria, Republic of Seychelles; i Division of Epidemiology and Biostatistics, School of Public Health, University of Cape Town, Cape Town, South Africa; j School of Public Health, Boston University, Boston, MA, USA; k Section of Endocrinology, Diabetes, and Metabolism, Jesse Brown Veterans Affairs Medical Center, Chicago, IL, USA

**Keywords:** Toxic metals, Gut microbiome, Obesity, Type 2 diabetes, Cardiometabolic risk, African-origin populations

## Abstract

Underlying mechanisms by which exposures to toxic metals/metalloids impact obesity and type 2 diabetes (T2DM) risk remain largely unknown. Gut microbiota have been strongly associated with cardiometabolic risk. To assess relationships between high metal exposures, gut dysbiosis, and metabolic dysregulation, we analyzed associations among gut microbiome taxa, dichotomized metal levels (arsenic, lead, mercury, cadmium), clinical measures (BMI, fasting blood glucose, blood pressure), and diagnoses (hypertension, obesity, diabetes) in 178 African-origin adults (52% female, mean age = 43.0 ± 6.4 years) from Ghana, South Africa, Jamaica, Seychelles, and USA. High vs. low lead and arsenic levels had a significant effect on beta diversity (*p* < 0.05). Seventy-one taxa were associated with high lead levels: 30 with elevated BMI, 22 with T2DM, and 23 with elevated fasting blood glucose (*p* < 0.05); 115 taxa were associated with high arsenic levels: 32 with elevated BMI, 33 with T2DM, and 26 with elevated blood glucose (*p* < 0.05). Porphyrin metabolism was the most enriched metabolic pathway in taxa associated with higher lead and arsenic exposure. These data provide the first findings from African-origin adults that demonstrate the association between the gut microbiome with lead and arsenic exposure and obesity and T2DM risk.

## Introduction

Type 2 diabetes mellitus (T2DM) and obesity are increasing worldwide health challenges associated with significant disease burden, comorbidities, and healthcare costs.^20,29,46^ Globally, it is projected that 2.7 billion adults will be overweight or obese by 2025, and between 2025 and 2045, people with T2DM will increase by 212 million to 783 million.^29,46^ By 2045, Northern Africa, currently highest in regional T2DM prevalence at 16.2%, is expected to see the number of individuals with diabetes increase to 136 million people, while sub-Saharan Africa is projected to have the highest T2DM prevalence increase of 129% to 55 million people.^29^ Black Americans are disproportionately affected by obesity and T2DM, contributing to significant health disparities in the US.^1^ While obesity is a main driver of T2DM, both obesity and T2DM also increase the risk of other highly prevalent cardiometabolic diseases (CMDs).^26^ Successful strategies for management and treatment of obesity and T2DM require a more complete understanding of the risk factors that drive the complex heterogeneous etiopathology of these diseases.

Mounting evidence suggests environmental exposures, including to toxic metals/metalloids (hereafter, “metals”), may contribute to CMD risk.^20,27,31,32,41,46,48,52,57^ Metal (arsenic, cadmium, lead, and mercury) exposures come from food, water, and airborne sources.^20,27,31,32,36,46,52,57^ For example, gold mining in Ghana has polluted river water with metals.^5^ Lead, mercury, cadmium, and arsenic induce organ dysfunction even with low level exposures.^30,43,64^ Arsenic, lead, and cadmium have been linked to increased risk of elevated fasting blood glucose (FBG), and arsenic has been linked to increased diabetes prevalence and worsening glycemic control.^32,59^ Although multiple mechanisms have been proposed, especially for arsenic, no specific mechanism clearly explains these associations.^20,46^

The gut microbiota, including their composition and microbially produced metabolites, are increasingly thought to be significant players in the development and progression of obesity and T2DM.^17,19,23,26,28,35,45,54,55^ The degree of dysbiosis appears to be associated with both obesity and T2DM disease severity.^17,19,28,35,45,54,55^ In animal models, fecal microbiota transfer from individuals with T2DM or obesity can replicate the disease phenotype.^19,35,45,54,55^ Critically, this relationship between T2DM or obesity and the gut microbiome has been suggested to be bidirectional.^11^ Overall, gut microbiota have been linked to metabolic disease development by multiple different mechanisms, including inflammation, gut barrier integrity, and microbial metabolites that act as signaling molecules.^23^

Recent evidence in animal models indicates that metal exposures may be linked to gut microbiome dysfunction; however, little data is available from human studies.^16^ In those animal studies, metals have been associated with gut dysbiosis through altered microbial composition that subsequently impacts host physiologic processes and metabolic functions of the gut microbiome.^3,7,12,14,24,31,37,50,58^ Lead, cadmium, and arsenic exposure have each been associated with decreased microbial diversity and specific differentially altered genera.^3,7,12,14,24,36,37,50^ Metal exposures are also linked to altered metabolism of vitamins, bile acids, and other biomolecules and cofactors, where key steps in production of active biomolecules occur through microbial metabolism impacting host metabolism.^7,12,14,24,36,37,50,58^ With human exposure best characterized as mixtures, the available data suggest that co-exposure to multiple metals have also been linked to increased T2DM and obesity risk relative to exposure to single metals,^56,61,63^ potentially via additive, synergistic, or antagonistic effects on the gut microbiota. As individuals are exposed to multiple metals at the same time, there is a growing concern about the health hazards posed by these co-exposures.^62^ Importantly, however, changes to the gut microbiota have primarily been studied for single or bi-metal exposures^8,21,44^ with recent studies of multiple metals exposures demonstrating similar changes to microbiome diversity as those seen in single metal exposures.^6^ Understanding the impact of metal exposures on the human gut microbiota and critically, how this influences cardiometabolic health, is urgently needed.

To our knowledge, this study is the first exploration of the association between toxic metal exposures, gut microbiota, and CMD risk in adults of African descent from five countries across the epidemiologic transition. We analyzed the difference between individuals with high versus low metal exposures across all study sites, as well as within sites. We hypothesized that high metal exposures and gut dysbiosis promote metabolic dysregulation and increase CMD risk. These data reveal evidence of important associations between metal exposures and the gut microbiome and, in turn, associations with the prevalence of obesity and T2DM in critically impacted human populations.

## Materials and methods

### Study population

The original study population consists of an international cohort of 2,506 African-origin adults with ~ 500 participants from each site: Ghana, South Africa, Jamaica, Seychelles, and USA. Participants are followed through the Modeling the Epidemiologic Transition Study (METS) (NIH R01-DK080763) and the currently funded METS-Microbiome study (NIH R01-DK111848).^18,38^ METS-Microbiome is examining the relationship between gut microbiota and obesity and T2DM risk.^18^ METS-Microbiome collects urine samples, stool for gut microbiota sequencing, and clinical labs for measures of cardiometabolic health, which include T2DM risk (fasting insulin and glucose levels), cardiovascular risk (lipids), and kidney function (spot urine creatinine/albumin ratio).^18,38^

The five METS research sites were selected as representative of the epidemiologic transition continuum, which is a model that captures the transition from predominantly infectious diseases to non-communicable diseases that accompany increasing economic development.^18,38,42^ The epidemiologic transition framework is defined using the United Nations Human Development Index (HDI) to study health outcomes across all sites with Ghana representing lower-middle-income, South Africa representing middle-income, Jamaica and Seychelles representing high-income, and the US representing very high-income.^18,38^

Individuals were excluded from participating in the original METS study if they self-reported being persons with an infectious disease, including HIV, being pregnant, breastfeeding, or having any condition that prevented the individual from participating in normal physical activities.^18,38^ Participants were further excluded from METS-Microbiome if they self-reported using antibiotics in the preceding three months. Descriptions of both the METS and METS-Microbiome protocols for data collection, measurement, and laboratory procedures have been published.^18,38^ Both METS and METS-Microbiome studies were individually approved by the Institutional Review Board of Loyola University Chicago, IL, US, which is the coordinating center.^18,38^ For international sites, the protocols were approved by their respective institutions.^18,38^ All study procedures were explained to participants in their local languages, and participants were provided written informed consent after being given the opportunity to ask any questions and compensated for their participation.

### Urinary metals and microbiome assessments

As part of the METS-Microbiome protocol, urine samples were obtained in 2019, from which 178 samples were randomly selected and analyzed for metal levels (Table 1). These included concentrations of arsenic, cadmium, lead, and mercury. To assess the impact of metals exposure on the microbiota in this subset, we accessed the 16S rRNA sequence data from 2019 and the measured clinical phenotypes to evaluate the most significant associations.

**Table 1.** Characteristics of study participants for all sites. Aggregate data of metal exposure and cardiometabolic risk factors by site and across the dataset. Count and percentage of the population reported for categorical variables. Mean and standard deviation are reported for continuous variables.

	Ghana (*N* = 30)		South Africa (*N* = 34)		Jamaica (*N* = 38)	Seychelles (*N* = 40)	US (*N* = 36)	Overall (*N* = 178)
**Age (years)**, Mean (SD)	42 (± 6.1)		40 (± 6.8)		42 (± 7.0)	44 (± 5.3)	45 (± 6.3)	43 (± 6.4)
**Sex, n (%)**								
Men	12 (40%)		15 (44%)		19 (50%)	20 (50%)	19 (53%)	85 (48%)
Women	18 (60%)		19 (56%)		19 (50%)	20 (50%)	17 (47%)	93 (52%)
**Obesity, n (%)**								
Obese	16 (53%)		16 (47%)		19 (50%)	20 (50%)	19 (53%)	90 (51%)
Non-obese	14 (47%)		18 (53%)		19 (50%)	20 (50%)	17 (47%)	88 (49%)
**BMI (kg/m**^**2**^), Mean (SD)	30 (± 12)		32 (± 14)		32 (± 14)	31 (± 12)	36 (± 15)	32 (± 14)
**Diabetes, n (%)**								
Diabetic	3 (10%)		2 (6%)		2 (5%)	8 (20%)	7 (19%)	22 (12%)
Non-diabetic	27 (90%)		32 (94%)		36 (95%)	32 (80%)	29 (81%)	156 (88%)
**Glucose (mg/dL)**, Mean (SD)	110 (± 22)		96 (± 24)		100 (± 24)	100 (± 24)	110 (± 65)	100 (± 38)
**Hypertension, n (%)**								
Hypertensive	11 (37%)		16 (47%)		21 (55%)	21 (52%)	24 (67%)	93 (52%)
Non-hypertensive	19 (63%)		18 (53%)		17 (45%)	19 (48%)	12 (33%)	85 (48%)
**Arsenic Exposure, n (%)**								
Low	6 (20%)		25 (74%)		17 (45%)	7 (18%)	33 (92%)	88 (49%)
High	24 (80%)		9 (26%)		21 (55%)	33 (82%)	3 (8%)	90 (51%)
**Lead Exposure, n (%)**								
Low	5 (17%)		16 (47%)		25 (66%)	14 (35%)	28 (78%)	88 (49%)
High	25 (83%)		18 (53%)		13 (34%)	26 (65%)	8 (22%)	90 (51%)
**Cadmium Exposure, n (%)**								
Low	14 (47%)		23 (68%)		15 (39%)	16 (40%)	18 (50%)	86 (48%)
High	16 (53%)		11 (32%)		23 (61%)	24 (60%)	18 (50%)	92 (52%)
**Mercury Exposure, n (%)**								
Low	12 (40%)		28 (82%)		17 (45%)	1 (2%)	27 (75%)	85 (48%)
High	18 (60%)		6 (18%)		21 (55%)	39 (98%)	9 (25%)	93 (52%)

Values provided as mean and standard deviation or number and percentage; Metal exposures calculated as high and low relative to the population median value.

### Anthropometry, sociodemographic, and biochemical measurements

METS visits were conducted at community-based research clinics within the respective communities, early in the morning following an overnight fast. Weight (kg) and height (m) measurements were captured using standard techniques and used to calculate BMI as kg/m^2^. Obesity was defined as a BMI >30 kg/m^2^. Participants provided a blood sample, from which fasted blood glucose (FBG) was measured; insulin, leptin, and adiponectin were measured using radioimmunoassay kits (Linco Research, Inc., St. Charles, MO). T2DM was defined as FBG >125 mg/dL or by current treatment; however, among participants from Ghana, because not all the participants were overnight fasted, presumed T2DM was defined as glucose ≥140 mg/dL or current treatment using the American Diabetes Association guidelines for random glucose testing.^18,38^ Blood pressure (BP) was measured in triplicate at two timepoints during each examination using an automatic digital monitor (model HEM-747Ic, Omron Healthcare, Bannockburn, IL, USA). Hypertension was defined as BP > 130/80 mmHg or on current antihypertensive treatment. Spot urine samples were also assayed for urinary albumin and creatinine levels.

### Urinary metals quantification and stratification

Urine samples were analyzed for arsenic, cadmium, lead, and mercury using inductively coupled plasma mass spectrometry (ICP-MS). Urinary creatinine was measured using a method based on the Jaffe reaction for standardizing metal concentrations to control for kidney function and hydration status.^51^ The core laboratory participates in the Quebec Multielement External Quality Assessment Scheme of the Institut National de Santé Publique du Québec, Canada for external accuracy assessment of biological samples. To increase power in assessing metal effects, each metal was stratified by median metal levels across all sites (the study population median lead = 0.86ug/g, median arsenic = 25.6ug/g, median cadmium = 0.39ug/g, and median mercury = 0.34ug/g) for a between-site metals exposure level.

### DNA extraction and amplicon sequencing

Fecal samples were shipped to the Microbiome Core sequencing facility (University of California, San Diego, UCSD) and randomized for 16S rRNA gene processing to extract DNA with MagAttract Power Microbiome kit using blank controls and ZymoBIOMICS mock controls (Cat. No. D6300) in each extraction plate.^18,19^ The V4 region of 16S rRNA gene was amplified from extracted DNA with 515F-806 R region-specific primers according to the Earth Microbiome Project.^53^ Purified amplicon libraries were sequenced on the Illumina platform to produce 150 bp forward and reverse reads (IGM Genomics Center at UCSD).^18,19^

### Bioinformatic analysis

We analyzed microbiota amplicon data using existing pipelines to identify taxonomic markers for all samples. In Qiita, generated raw sequence data was demultiplexed, quality filtered, and trimmed.^18,19,25^ Amplicon Sequence Variants (ASVs) were defined using DeBlur, and taxonomy was assigned using a Naïve-Bayes classifier compared against a SILVA (version 138) reference database.^19,40^ Microbiota samples were matched to study participants for whom phenotype, clinical and urine metal metadata were available. The resulting ASV abundance count table, taxonomy data, and sample metadata were exported and merged into a phyloseq object in R (R Foundation for Statistical Computing, Vienna, Austria) for downstream analysis.^19^ The phyloseq object was then used for quality control to remove: 1) ASVs with less than ten reads in the entire dataset and samples with fewer than 5000 reads; 2) ASVs that were unassigned at the phylum level; and 3) ASVs with fewer than 50 reads across all samples or were in less than 2% of samples. Quality control resulted in 16,399 ASVs with 9264 genus-level taxa across 178 participants.

### Alpha and beta diversity analysis

Microbial alpha diversity was measured using the Shannon index via the microbiome library.^19^ Beta diversity was calculated with pairwise Bray-Curtis dissimilarity, and significance was calculated with permutational multivariate analysis of variance (PERMANOVA) using phyloseq.^19^ Univariate comparisons were performed in two-sample two-tailed t-tests when we could assume normality, and Wilcoxon Signed Rank tests when we could not. Benjamini-Hochberg (BH) adjusted p-values of less than 0.05 were considered statistically significant.

### Linear discriminant analysis

Linear discriminant analysis (LDA) effect size was performed on per-sample normalized relative abundances.^49^ This algorithm estimates microbial taxa that contribute to observed differences by metal exposures.^49^ We evaluated differences by coupling a univariate non-parametric test (Wilcoxon rank-sum, α = 0.05) with LDA scores (threshold for discriminative features > 3.0) to calculate effect size of identified differentially abundant taxa stratified by each individual categorical metal exposure metadata.

### Differential abundance association analysis of microbial taxa

To identify multivariable associations within these data, four linear mixed effect models were used with individual taxa, urinary metal levels, and cardiometabolic risk profiles with MaAsLin2.^39^ The ASV abundance table was transformed with trimmed mean of *m* values and run with a linear mixed model.^39^ For each metal, the model measured individual associations {taxa ~ metal + cardiometabolic variables + confounders}. A final model, to analyze the influence of all metals, included four metal and cardiometabolic variables {taxa ~ arsenic + lead + cadmium + mercury + cardiometabolic variables + confounders}. The cardiometabolic variables included obesity, T2DM, and hypertension diagnosis, with blood glucose levels, systolic and diastolic BP, and BMI. Each model controlled for site, sex, and age as confounders. From these four models, associations are calculated between taxonomic relative abundance at the genus level, metal exposures, and cardiometabolic variables. P-values were corrected for multiple comparisons using the BH correction, α = 0.05.

### Predicted metabolic gene pathway analysis

Genera significantly associated with metals exposure were used to predict (q-value <0.05 to reduce false discovery rate) functional metabolic pathways. We utilized the Phylogenetic Investigation of Communities by Reconstruction of Unobserved Species 2 (PICRUSt2) v2.5.1.^15^ Normalized ASV abundance table and weighted nearest-sequenced taxon index values per sample were used for predicting Enzyme Commission numbers and annotated using the MetaCyc database to identify enriched metabolic pathways.^15^ The resulting enriched metabolic pathway abundances were visualized. P-values were adjusted for multiple comparisons using the BH correction, α = 0.05.

## Results

We analyzed data from 178 METS Microbiome study participants (median age = 43.0 ± 6.4 years, 52% women) (Table 1). Median (IQR) BMI was lowest in Ghana, 20.82 kg/m^2^ (19.08, 38.36), and highest in the US, 30.12 kg/m^2^ (22.63, 49.26). 14.3% of the total study population had T2DM, with the highest (20%) in the US and Seychelles and the lowest (5%) in Jamaica. The highest lead levels were in Ghana, median (IQR) = 1.36 µg/g (1.11, 1.76) (83% had elevated levels), and lowest in US, 0.53 µg/g (0.38, 0.75) (22% had elevated levels). Arsenic levels were highest on average in Ghana; however, more participants had high arsenic exposure in Seychelles. In Ghana, median (IQR) arsenic level was 72.96 µg/g (39.5, 108.09) (80% had elevated levels) and in Seychelles, median (IQR) arsenic level was 61.71 µg/g (41.15, 97.0) (82% had elevated levels). Arsenic levels were lowest in the US at 7.99 µg/g (6.01, 12.66) (8% had elevated levels). Cadmium levels were highest in Jamaica, 0.508 µg/g (0.29, 0.90) (61% had elevated levels), and lowest in South Africa, 0.25 µg/g (0.16, 0.41) (32% had elevated levels). Mercury levels were highest in Seychelles, 1.98 µg/g (1.38, 2.54), (98% had elevated levels) and lowest in South Africa, 0.064 µg/g (0.007, 0.18), (18% had elevated levels) (Table 1).

### Alpha diversity

**Figure 1.**
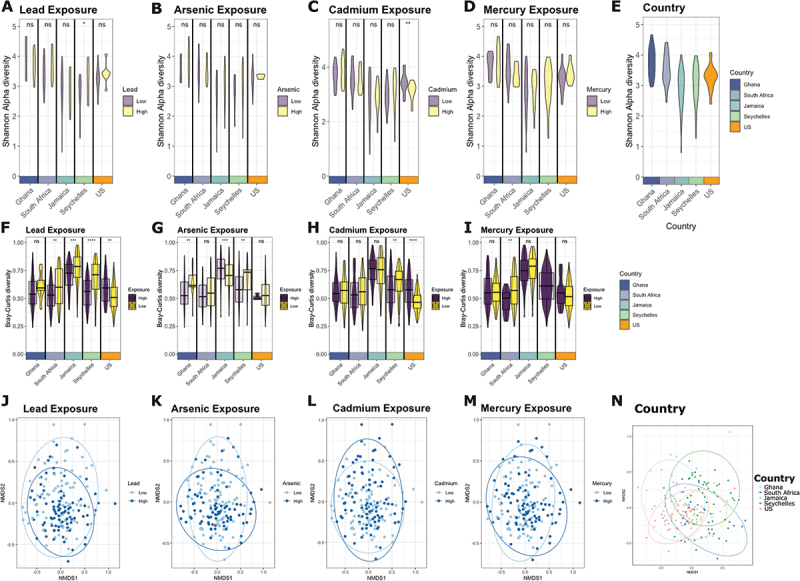
A-E) Shannon alpha diversity by metals exposure between countries of origin. Alpha diversity was significantly distinct by A) high lead exposure in Seychelles and C) high cadmium exposure in the US, but no significance existed by site with B) high arsenic exposure and D) high mercury exposure. E) Alpha diversity across sites. Alpha diversity is highest in Ghana and South Africa, and decreases in Jamaica, Seychelles, and USA. All associations are significant except between Jamaica and the US, Ghana and South Africa, and Jamaica and Seychelles. F-I) Beta diversity by metals exposure between countries of origin. F) High vs low lead exposure was significant for South Africa, Jamaica, Seychelles, and USA. G) High vs low arsenic exposure was significant for Ghana, Jamaica, and Seychelles. H) High cadmium exposure was significantly different from low exposure for Seychelles and USA. I) High vs low mercury exposure was significant for South Africa. Beta diversity was measured by the Bray Curtis dissimilarity metric. J-N) Beta diversity across all countries. Across all sites, J) high vs low lead exposure and K) high vs low arsenic exposure were significantly different by PERMANOVA (p-value < 0.05) when controlling for country of origin. L) High vs low cadmium exposure and M) mercury exposure were not significant by PERMANOVA (p-value < 0.05) when controlling for country of origin. N) Country beta diversity across sites. By PERMANOVA, there are significant differences (p-value < 0.05) between Ghana and all other sites, South Africa and Seychelles, South Africa and USA, Seychelles and USA, Seychelles and Jamaica, and USA and all other sites. ****adjusted *p* < 0.0001, ***adjusted *p* < 0.001, **adjusted *p* < 0.01, *adjusted *p* < 0.05, ns *p* > 0.05, paired Wilcoxon test.

Gut bacterial diversity and richness measured by the Shannon alpha diversity index varied by country of origin, being higher in Ghana and South Africa, and lower in Seychelles, Jamaica, and the US (Figure 1E). However, microbial alpha diversity and richness between high versus low metal levels were only significant for high lead exposure in Seychelles and high cadmium exposure in the US (Figure 1A-D). Overall, metals in our cohort had a minimal effect on alpha diversity.

### Beta diversity

Differences in bacterial composition or beta diversity demonstrated Ghana and the US were both significantly different from all other sites, and Seychelles was significantly different from South Africa and Jamaica (Figure 1N). Comparing high to low metal exposure groups, beta diversity was significantly different for lead and arsenic (Figure 1J-M). By site, beta diversity was significantly lower in the high lead exposure group from South Africa, Jamaica, and Seychelles and higher in the US (Figure 1F). In the high arsenic exposure group, beta diversity was lower in Ghana and Seychelles and higher in Jamaica (Figure 1G). For the high cadmium group, beta diversity was lower in Seychelles and higher in the US (Figure 1H). For the high mercury group, beta diversity was lower in South Africa (Figure 1I).

### Linear discriminant analysis

**Figure 2.**
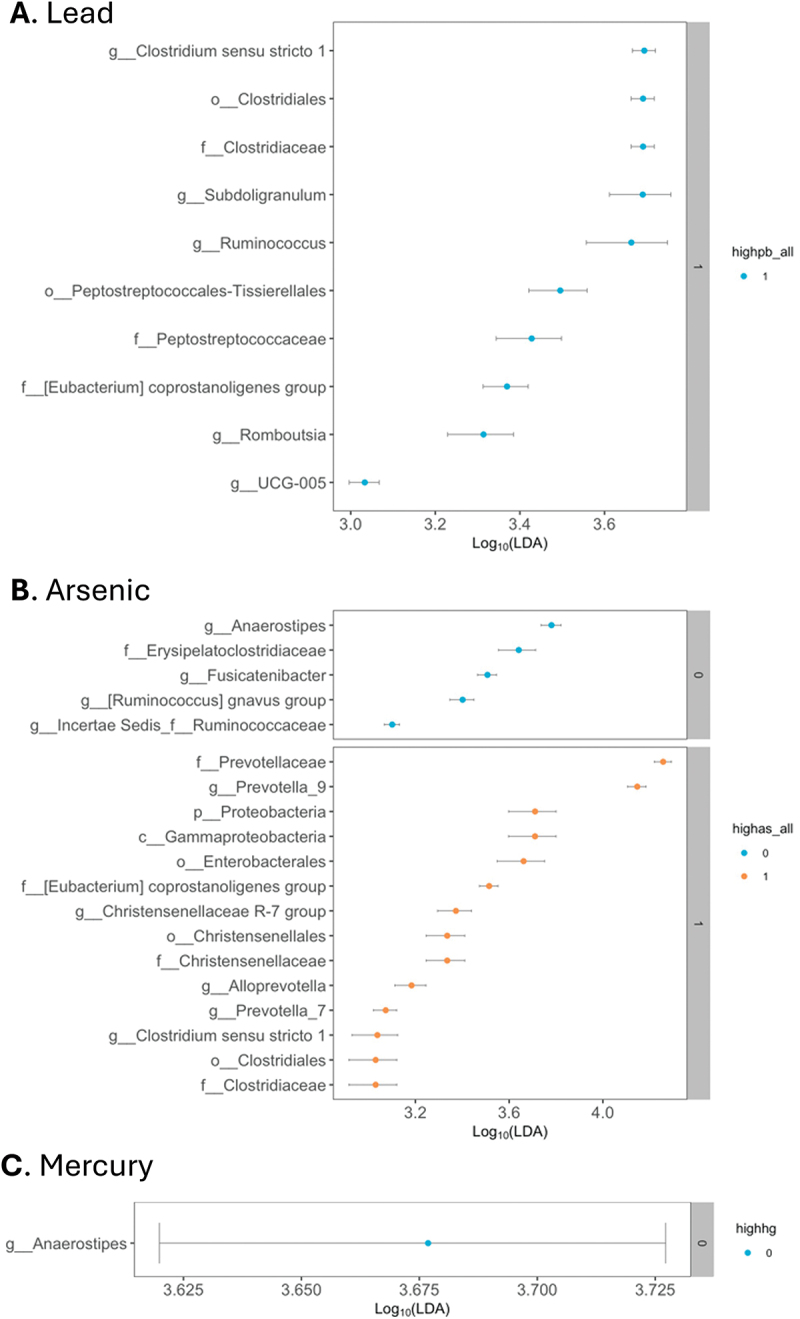
Differential taxa by metal exposure. A) High lead exposure characterized by several taxa. Low lead exposure was not characterized by any taxa. Lead denoted as “highpb_all” with high lead categorized as “1”; low as “0”. B) High arsenic-exposed communities were unique compared with lower exposure. Arsenic is “highas_all” with high arsenic categorized as “1”; low as “0”. C) Low mercury-exposed microbial communities were only differentially characterized by one taxon. High mercury exposure was not characterized by any taxa. Mercury is “highhg” with high mercury categorized as “1”; low “0”.

With high lead exposure, *Clostridium*, *Subdoligranulum*, *Ruminococcus*, and *Peptostreptococcales* were the most differentially abundant taxa (Figure 2A). With low lead exposure, no taxa were differentially abundant (Figure 2A). *Firmicutes* and *Proteobacteria* were the primary phyla differentially abundant in high arsenic exposed microbial communities (Figure 2B). The taxa overrepresented with high arsenic exposure included *Prevotella*, *Proteobacteria*, *Gammaproteobacteria*, *Enterobacterales*, *Christensenellaceae*, *Alloprevotella*, and *Clostridiales* (Figure 2B). With low arsenic exposure, differentially abundant taxa included *Anaerostipes*, *Erysipelatoclostridiaceae*, *Fusicatenibacter*, and *Ruminococcus* (Figure 2B). Mercury-exposed microbial communities were only differentially characterized by *Anaerostipes* (Figure 2C). High lead and both low and high arsenic were associated with differential regulation of associated taxa.

### Lead exposure

**Figure 3.**
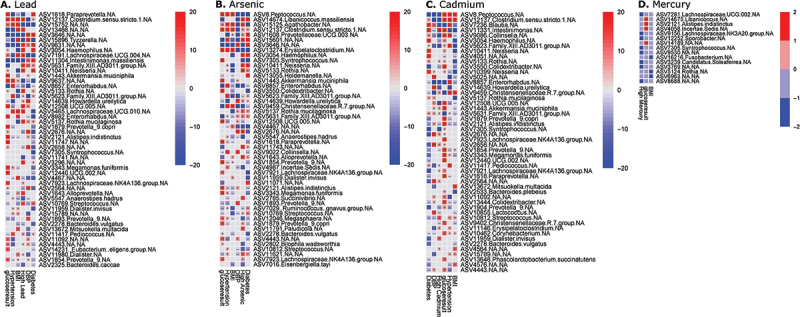
Differential taxa by individual metal exposure and cardiometabolic disease (CMD) factors. A) Lead and CMD factors, B) arsenic and CMD factors, C) cadmium and CMD factors, and D) mercury and CMD factors. In each linear mixed model, taxa associated with metal exposure, diabetes diagnosis (diabetes), obesity diagnosis (obesity), hypertension diagnosis (hypertension), fasting glucose result (glucoseresult), BMI (BMI), systolic blood pressure (SBP), and diastolic blood pressure (DBP). Significance was calculated by $-\log\left(\mathrm{qval}\right)*\mathrm{sign}\left(\mathrm{coeff}\right)$.

**Figure 4.**
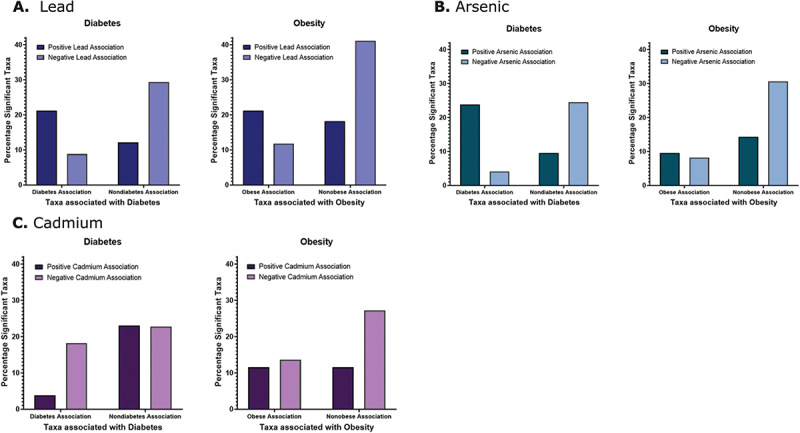
Percentage of significant taxa jointly associated with individual high metal exposure and obesity or T2DM. A) Taxa positively associated with lead exposure jointly associated with obesity and T2DM. More lead-associated taxa were positively associated with T2DM (21% of taxa) than negatively associated (12%) (dark blue). More lead-associated taxa also were positively associated with obesity (21%) than negatively associated (18%) (dark blue). Taxa that decreased abundance with high lead exposure were positively associated with a healthy phenotype and negatively associated with obesity (41%) and T2DM (29%) (light blue). B) More arsenic-associated taxa were positively associated with T2DM (24%) than negatively (10%). Ten percent of taxa were positively associated with obesity and 14% negatively (dark green). Of the taxa that decreased with high arsenic exposure, more positively associated with a healthy phenotype and negatively associated with obesity (31%) and T2DM (24%) (light green). C) Of the taxa that increased with high cadmium, 4% were positively associated with T2DM and 23% negatively associated (maroon). Twelve percent of taxa were positively associated with obesity and 11% negatively associated (maroon). Of the taxa that decreased with high cadmium, 23% were negatively associated with T2DM and 27% negatively associated with obesity (pink).

A total of 71 genera were significantly associated with high lead exposure: 30 strongly associated with BMI, 23 with FBG, and 22 with T2DM. Of these genera, 33 were significantly higher and 34 were significantly lower in abundance with high lead exposure. High lead exposure was associated with higher phyla *Bacteroides* and lower *Firmicutes*. At the genus level, high lead exposure was significantly associated with *Paraprevotella*, *Clostridium*, *Tyzzerella*, *Haemophilus*, and *Lachnospiraceae* (Figure 3A). High lead exposure was associated with higher BMI (Figure 3A) and increased obesity and T2DM prevalence (Figure 4A). For lead-associated taxa, BMI positively associated with *Clostridium*, *Lachnospiraceae*, *Tyzzerella*, *Alloprevotella, and Dialister*, and negatively associated with *Haemophilus*, *Neisseria*, *Rothia, Streptococcus*, and *Family XIII AD3011 group*. Lead-associated taxa positively associated with T2DM included *Clostridium*, *Haemophilus*, *Neisseria*, *Streptococcus, Family XIII AD3011 group*, and *Pediococcus* (Figure 3A). Significant associations were found between high lead exposure, BMI, and hypertension for all lead-associated taxa, excluding *Haemophilus*, which associated only with high lead exposure and T2DM (Figure 3A).

Twenty-one percent of taxa were positively associated with the high lead group and being obese, compared to 18% associated with a normal weight phenotype. Of the lead-associated taxa, 21% were also associated with T2DM diagnosis compared to 12% with a non-diabetic phenotype (Figure 4A). For taxa with a lower abundance in the high lead exposed group, 41% were associated with a non-obese phenotype (vs. 12% with obesity) and 29% were associated with non-diabetic phenotype (vs. 9% with T2DM) (Figure 4A). Thus, high lead exposure resulted in an increased abundance in taxa associated with obesity and T2DM, and decreased abundance of taxa associated with nonobese and non-diabetic traits (Figure 4A).

### Arsenic exposure

A total of 115 taxa were significantly associated with high arsenic exposure: 32 with BMI, 26 with FBG, and 33 with T2DM. Of these genera, 63 were significantly higher, and 49 were significantly lower. High arsenic exposure was correlated with an increased abundance of taxa associated with T2DM (Figures 3B and 4B). Genera that had the most significant positive association with high arsenic exposure were *Libanicoccus*, *Agathobacter*, *Haemophilus, Neisseria*, *Alloprevotella*, *Succinivibrio*, and *Rothia*. Arsenic was significantly associated with T2DM, and both high arsenic exposure, and T2DM were significantly associated with multiple taxa, but most significantly with *Peptococcus*, *Libanicoccus*, *Agathobacter*, *Clostridium*, *Prevotellaceae*, *Haemophilus*, *Syntrophococcus*, *Neisseria*, and *Rothia* (Figure 3B). *Clostridium, Prevotellaceae*, and *Alloprevotella* were positively associated with both T2DM and BMI. *Neisseria*, *Haemophilus*, and *Rothia* were positively associated with T2DM and negatively associated with BMI. Finally, *Agathobacter* was shown to be positively associated with BMI and FBG, while negatively associated with T2DM (Figure 3B).

### Cadmium exposure

Forty-eight genera were significantly associated with high cadmium exposure, with 26 positively associated and 22 negatively associated with exposure. Of the cadmium-associated genera, 14 were associated with BMI, 15 with FBG, and 15 with T2DM. The top genera that had the most positive association were *Intestinimonas*, and negative associations were *Peptococcus*, *Clostridium*, *Blautia*, *Collinsella*, *Enterorhabdus*, *Howardella*, and *Syntrophococus* (Figure 3C).

High cadmium exposure was associated with lower abundance of taxa associated with a nonobese or non-diabetic phenotype. Thus, high cadmium exposure was associated with obesity and T2DM (Figure 4C). Of the top cadmium-associated genera, *Peptococcus* and *Clostridium* were positively associated with BMI while the rest were negatively correlated. *Clostridium*, *Blautia*, *Intestinimonas*, and *Collinsella* were positively correlated with T2DM, and *Peptococcus* and *Syntrophococcus* were negatively associated. High cadmium exposure and T2DM were significantly positively associated with *Intestinimonas, Rothia*, and *Family XIII AD3011 group;* the high cadmium group and T2DM were negatively associated with *Peptococcus, Pediococcus, Colidextribacter*, *Enterorhabdus*, and *Syntrophococcus* (Figure 3C).

### Mercury exposure

Twelve taxa were significantly associated with high mercury exposure, though none were jointly associated with any cardiometabolic variables (Figure 3D).

### Comparing all metal exposures

**Figure 5.**
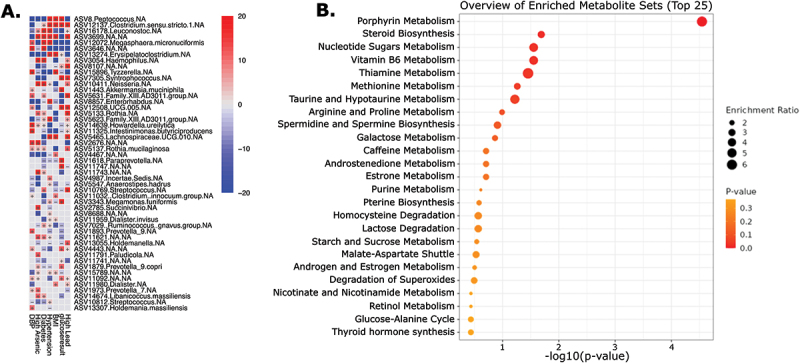
A) Top 50 taxa associated with all high metal exposures (lead, arsenic, cadmium, and mercury) and cardiometabolic factors, controlling for site, sex, and age. For the linear mixed model, taxa were associated with high lead exposure (high lead), high arsenic exposure (high arsenic), high cadmium exposure (high cadmium), high mercury exposure (high mercury), diabetes diagnosis (diabetes), obesity diagnosis (obesity), hypertension diagnosis (hypertension), fasting glucose result (glucoseresult), BMI (BMI), systolic blood pressure (SBP), and diastolic blood pressure (DBP), while controlling for sex, age, and site. Significance was calculated by -log(qval)*sign(coeff). B) Top functional metabolic pathways. Porphyrin metabolism was the most enriched metabolic pathway in the taxa associated with metal exposure. Steroid biosynthesis, nucleotide sugars metabolism, vitamin B6 metabolism, thiamine metabolism, and methionine metabolism were all significantly enriched in this group (adjusted *p* value < 0.05).

To understand how all metal exposures are associated with obesity and T2DM, we next ran a linear mixed model of all metal exposures and outcomes of interest to identify microbial taxa associated with multiple high metal exposures and clinical metabolic variables. *Peptococcus*, *Leuconostoc*, *Erysipelatoclostridium*, *Haemophilus*, *Tyzzerella*, and *Neisseria* significantly correlated with high lead exposure and high arsenic exposure, as well as T2DM. Additionally, high lead exposure alone positively correlated with BMI and had significant positive associations with *Clostridium* and *Megasphaera micronuciformis*. High arsenic exposure strongly correlated with 40% of the taxa significantly associated with T2DM. (Figure 5A)

The results of this model demonstrated multiple genera significantly associated with high lead or high arsenic exposure and jointly associated with clinical measures of obesity and T2DM. High lead exposure was most strongly associated with abnormal FBG and elevated BMI, while high arsenic exposure was associated with T2DM and elevated diastolic BP. These results match with findings from previous mixed models for each individual metal (Figures 3A-D and 4A-C), which showed high lead exposure associated most closely with obesity and high arsenic exposure associated with T2DM. (Figure 5A)

### Metabolic pathways

From the microbes associated with metal exposure, high BMI, and increased FBG, we found that porphyrin metabolism was the most highly enriched metabolic pathway and most significant by p-value (adjusted *p* < 2.1 × 10^−16^). Additionally, steroid biosynthesis, nucleotide sugars metabolism, vitamin B6 metabolism, thiamine metabolism, and methionine metabolism were all significantly enriched in this group (adjusted *p* < 0.05). (Figure 5B)

## Discussion

Previously, lead and arsenic have been shown to be associated with obesity and T2DM, with high arsenic exposure associated with higher T2DM prevalence and glycemic deterioration, as demonstrated in both human and animal studies.^16,20,27,31,32,41,46,48,52,57,59^ Exposure to metals has previously been shown to have detrimental effects on the composition of the gut microbiota by both enhancing deleterious and suppressing beneficial taxa; however, most of these studies were performed in mouse models.^3,7,12,14,24,31,36,37,50^ A limited number of observational and retrospective studies have examined either arsenic or lead effects on the human gut microbiome, though none examined links to cardiometabolic outcomes.^7,14,50^ In mice, lead and arsenic have been shown to disturb gut microbiota by decreasing diversity.^24^ As gut microbiota have been associated with metabolic risk, we assessed the linkage between toxic metals exposures and cardiometabolic outcomes in a diverse African-origin cohort. We found that two metals, lead and arsenic, are substantially associated with bacterial composition as well as T2DM and obesity. Notably, the results from this study suggest that this increased risk may be mediated by microbial alterations to the bacterial porphyrin pathway.

Regarding possible mechanisms for these associations, we found that arsenic depletes commensal bacteria in *Firmicutes*, including *Ruminococcus* and *Erysipelatoclostridiaceae*, both of which exhibit reduced abundance in the high arsenic exposure group and greater abundance in the low arsenic group. Among those with high exposure, microbiota show greater abundance of *Prevotella*, *Christensenella*, and *Clostridium*. In the high lead-exposed group, *Clostridium*, *Peptostreptococcales*, *Subdoligranulum*, and *Ruminococcus* had greater abundance. High arsenic and high lead exposures each significantly correlated with increases in opportunistic taxa, including *Haemophilus*, *Tyzzerella*, and *Neisseria*, and decreased commensal taxa, including *Peptococcus*, *Leuconostoc*, *Erysipelatoclostridium*, and both metals were associated with increased T2DM. In sum, arsenic and lead exposure seem to differentially impact taxa, where these findings suggest that metals may increase the risk of obesity and T2DM by removing commensal taxa, possibly impacting metabolic protection provided through metabolites produced by the microbiota, and enriching taxa with pathogenic potential.^7,13,50^ The changes observed in the composition of the microbiota suggest that high metal exposure is associated with gut dysbiosis and may contribute to increased risks of obesity and T2DM.

We found that the metabolism of the metal exposed microbial community is enriched in porphyrin metabolic pathways relative to taxa not associated with metal exposure. Porphyrin with an iron cofactor comprises heme and acts as an electron shuttle for the electron transport chain and as a molecule modulating redox signaling and stress, both of which are critical in cellular respiration.^10,13,22,34^ Notably, porphyrin metabolism plays a fundamental role in bacterial physiology as iron is a required cofactor for bacterial enzymes and proteins. Most bacteria have incomplete heme metabolic pathways; therefore, they require available heme and iron which they acquire via receptors and chelators from other bacteria and/or the host.^22^ Toxic metal exposures have been shown to affect heme synthesis through competition with iron by decreasing iron transport, reducing iron availability, and binding to proteins in place of iron.^13^ Thus, exposure to toxic metals may impair heme synthesis and activity through a variety of mechanisms. With metal-mediated impairment in heme and iron availability in the gut lumen, bacteria upregulate porphyrin metabolic pathways, a process important to bacterial metabolism.^22,47^ Interestingly, alterations in porphyrin metabolism and increased porphyrin metabolites have been associated with the development of insulin resistance and metabolic syndrome.^4,60^ The upregulation of porphyrin metabolism in the metal-exposed microbial community has previously been linked to and may serve to further gut dysbiosis.^33^ While acknowledging that toxic metals impact porphyrin metabolism in the host, the increase in porphyrin metabolism in metal-exposed microbiomes may be an additional factor contributing to gut dysbiosis and associated obesity and T2DM risk.^4^ Elucidating the potential role of metal-mediated disruptions in bacterial porphyrin metabolism in the downstream effects of gut dysbiosis on obesity and T2DM risk as well as distinguishing these effects from direct impacts of metal-induced alterations of porphyrin metabolism in the host require further investigation.

### Limitations

In this study, we have a single urinary estimate of toxic metal exposures, which may not reflect chronic exposures. For most metals, urinary measurements are well accepted for exposure evaluation.^2^ While blood is generally utilized for evaluation of lead exposures, studies with blood and urine lead levels correlate with each other, and urinary lead levels are considered a reasonable assessment for epidemiological studies.^9^ Metal levels were binarized for a high vs. low comparison in our study, which does not capture any dose-response to metals exposure or non-monotonic dose responses. This study primarily analyzed single metal effects with a single mixed model to study multiple taxa associations with all metals. Future analysis of multiple metal interactions and metal mixtures may add to the understanding of metal effects on T2DM and obesity.^64^ For multiple analyses, we controlled for several potential confounders, but other measured covariates were not applied to the final models, including diet, exercise, and smoking, which may act as confounders for this data. Sex can influence the microbiome and has been associated with immune function and hormones; sex-specific assessment of the links between toxic metals, the gut microbiome, and cardiometabolic disease risk require further interrogation.^64^ Finally, this cross-sectional analysis is limited in its ability to infer causality; however, these data provide strong justification for future studies examining longitudinal changes in exposure, outcomes, and microbiota composition to better define cause-and-effect relationships between these parameters.

## Conclusion

Toxic metal exposures have been associated with obesity and T2DM previously, and in animal models, these associations have been suggested to be partly driven by changes in the gut microbiota. To the best of our knowledge, our study of African-origin individuals has demonstrated for the first time that metal-mediated cardiometabolic disease risk may arise through changes in the gut microbiota. These metal effects may act by increasing taxa that are positively associated with obesity and T2DM and by decreasing taxa that are negatively associated with obesity and T2DM. These data are critical as many countries are heavily impacted by deteriorating environmental quality. Conversely, these results may justify novel strategies targeting the microbiome as a potential means of mitigating adverse metabolic effects of toxic metals, which may decrease the risk of obesity and T2DM, particularly in highly-exposed populations. While exposure reduction is the cornerstone of environmental health interventions, interventions that can modulate the gut microbiome may help address the impact of unavoidable exposures.

## Data Availability

All microbiome data have been previously made available in conjunction with a separate study.^19^ 16 S rRNA gene sequence data have been deposited at the European Bioinformatics Institute site under the accession code (https://www.ebi.ac.uk/ena/browser/view/PRJEB63378). Sequencing data and processed tables are available through QIITA under study identifier 13,512. Derived data supporting the findings of this study are available from the corresponding author, Julianne Jorgensen: jjorge6@uic.edu, on request. The SILVA 16 S rRNA database used for alignment is available at https://data.qiime2.org/2022.2/common/silva-138-99-515-806-nb-classifier.qza. and the MetaCyc Databases are available at https://metacyc.org/. The clinical and metadata are available under restricted access due to privacy regulations of our cohort. Access can be obtained by request to the corresponding author, Julianne Jorgensen: jjorge6@uic.edu or Lara Dugas: ldugas@luc.edu.
