# Correction

**DOI:** 10.1080/29933935.2025.2500151

**Published:** 2025-05-12

**Article title**: Toxic metals impact gut microbiota and metabolic risk in five African-origin populations

**Authors**: Julianne A. Jorgensen, Candice Choo-Kang, Luyu Wang, Lina Issa, Jack A. Gilbert, Gertrude Ecklu- Mensah, Amy Luke, Kweku Bedu-Addo, Terrence Forrester, Pascal Bovet, Estelle V. Lambert, Dale Rae, Maria Argos, Tanika N. Kelly, Robert M. Sargis, Lara R. Dugas, Yang Dai, and Brian T. Layden

**Journal**: *GUT MICROBES REPORTS*

**DOI**: https://doi.org/10.1080/29933935.2025.2481442

This article was originally published online with errors within the in-text citations and reference list.

These errors have now been corrected online.

